# SVC Is a Marker of Respiratory Decline Function, Similar to FVC, in Patients With ALS

**DOI:** 10.3389/fneur.2019.00109

**Published:** 2019-02-28

**Authors:** Susana Pinto, Mamede de Carvalho

**Affiliations:** ^1^Faculdade de Medicina, Instituto de Fisiologia e Instituto de Medicina Molecular, Universidade de Lisboa, Lisbon, Portugal; ^2^Department of Community and Rehabilitation, Rehabilitation Medicine, Umeå University, Umeå, Sweden; ^3^Department of Neurosciences and Mental Health, Hospital de Santa Maria-Centro Hospitalar Lisboa Norte, Lisbon, Portugal

**Keywords:** amyotrophic lateral sclerosis, functional outcome, predictor, rate of progression, slow vital capacity

## Abstract

**Introduction:** Respiratory function is a critical predictor of survival in amyotrophic lateral sclerosis (ALS). We aimed to determine if slow vital capacity (SVC) is a predictor of functional loss in ALS as compared to forced vital capacity (FVC).

**Methods:** Consecutive ALS patients in whom respiratory tests were performed at baseline and 6 months later were included. All patients were evaluated with revised ALS functional rating scale (ALSFRS-R) and the respiratory tests, SVC, and FVC. Significant independent variables of functional decay were assessed by univariate Kaplan-Meier log-rank test and multivariate Cox proportional hazards model. A monthly decay not exceeding 0.92 in ALSFRS was considered as the time event.

**Results:** We included 232 patients (134 men; mean onset-age 59.1 ± 11.23 years; mean disease duration from first symptoms to first visit: 14.5 ± 12.9 months; 166 spinal and 66 bulbar onset). All variables studied declined significantly between the two evaluations (*p* < 0.001). FVC and SVC were strongly correlated at study entry (*r*^2^ = 0.98, *p* < 0.001) and FVC and SVC decays between first evaluation and 6 months after were the only significant prognostic variables of functional decay (*p* < 0.001).

**Conclusion:** FVC and SVC decay are inter-changeable in predicting functional decay in ALS. Pharmacological interventions reducing the decline rate of FVC and SVC can have a positive impact on the global functional impairment, with relevant implications for clinical trials' design and interpretation.

## Introduction

Functionality is the quality or state of being functional, of having a specific functional status, which is described by the activities and participation of an individual in accordance with his present anatomy, physiology, and psychology and influenced by personal and environmental factors (1).

In amyotrophic lateral sclerosis (ALS), functionality is usually assessed by the revised ALS functional rating scale (ALSFRS-R), initially proposed in 1995 by Cedarbaum and Stambler (2) The initial version (ALSFRS) was composed by 10 questions, each graded from 4 (no function abnormality) to 0 (total disability) with a maximal score of 40 (normal functionality). To compensate the disproportionate weight of the bulbar and limb functions of the original scale, the authors added two additional respiratory questions to the pre-existing one (3). ALSFRS-R comprises 12 questions with a maximal score of 48, corresponding to normal functionality in the three evaluated domains—bulbar, spinal, respiration. This scale is applied worldwide in the ALS Units and used in clinical trials.

Respiratory insufficiency (RI) and other associated respiratory complications, especially lower respiratory infections, are the main cause of death in ALS (4). International guidelines recommend the assessment of respiratory function in ALS patients at first visit and every 3 months thereafter (5). Forced (FVC) and slow (SVC) vital capacities are non-invasive conventional tests used to estimate respiratory function in ALS. Their results depend on age, gender, height, and ethnicity, in addition to the functional integrity of the inspiratory and expiratory muscles (6, 7). FVC is sensitive to detect hypoventilation in ALS (8, 9) and can be more sensitive in detecting diaphragmatic weakness when performed in supine (10). FVC has been extensively studied in ALS and predicts hypoventilation and survival (8, 11–16), the reason why its values are determinant to patients' eligibility for clinical trials. In addition, it predicts functional decline as measured by the Appel ALS scale (11). SVC has not been so extensively studied although a growing interest has led to its inclusion as a frequent outcome in different recent clinical trials. It is a predictor of survival (17) and of disease progression as assessed by the number of regions affected (18). In ALS clinics, patients usually perform either FVC or SVC but not both. In a recent study (19), we found that SVC and FVC are very strongly correlated and decline similarly in ALS (about 2%/month), also verified for patients with bulbar-onset and those presenting respiratory involvement. The strong correlation between the two tests is maintained in very spastic patients affected with primary lateral sclerosis. Furthermore, both tests are strongly correlated with other respiratory tests namely maximal inspiratory (MIP) and maximal expiratory (MEP) pressures, and moderately correlated with clinical scores, although this correlation is weaker for bulbar-onset patients (19). In a second study (20), we verified that both FVC and SVC are similarly independent predictors of survival, with a 1.05 increased probability of death for each 1% decay in their percentage of predicted value.

With the present study we aim to investigate if SVC and FVC are independent predictors of functional decay in ALS as assessed by ALSFRS.

## Patients And Methods

### Study Population

We included consecutive ALS patients (with definitive or probable disease accordingly to the revised El Escorial criteria) followed in our ALS Clinic in Lisbon from January 2000 to December 31st 2014, without ventilatory support at study entry and with recordings of SVC, FVC and ALSFRS-R at first visit and 6 months after.

Patients with other medical conditions, namely heart failure, anemia, history of thoracic surgery, asthma, and chronic obstructive pulmonary disease, were excluded. Patients with clinical signs of dementia and/or unable to cooperate with the respiratory tests were not recruited.

### Investigations

Respiratory tests were performed within 1 month after the first clinical visit (baseline, T1) and a mean of 6 months after (T2).

#### Clinical Evaluation

All patients were evaluated with ALSFRS and ALSFRS-R including the its subscores. The decay of ALSFRS-R (absolute value) between T1 and T2 was considered.

#### Respiratory Function Tests—SVC and FVC

For each patient, the respiratory function tests were performed with the same devices and by the same technicians, always using nose clips for nose occlusion and according to ATS/ERS guidelines (21). SVC and FVC were assessed in the sitting position, using a computer-based USB spirometer (microQuark®, Cosmed®) or standard Jäger equipment (Jäger® Masterlab®, and Jäger® Masterscreen®, Erich Jäger, GmbH, Würzburg, Germany). All measurements were performed by one of the authors (SP), using microQuark®, Cosmed®, and the same technician for the Jäger® equipment. The best of three satisfactory and consistent expiratory maneuvers, each obtained after a maximal inspiratory effort, was used for analyses. Predicted values (%) were used (22). Decay between T1 and T2 was calculated for SVC and FVC.

### Statistical Analysis

The primary endpoint of this study was to test the independent predictors of functionality in a longitudinal data set. Secondary endpoints included comparing the weight of FVC and SVC as functional predictors. A monthly decay not exceeding 0.92 in ALSFRS was considered as the time event. This cut-off is mathematically equivalent to 1.1 in ALSFRS-R, a very accepted cutoff to define fast progressors in recent clinical trials (23). The decay in ALSFRS, as well as in SVC and FVC was calculated in accordance with what is suggested by Kimura et al. (24).

The whole population and the spinal and bulbar-onset subgroups were studied. Kaplan-Meier log-rank test and the multivariate Cox proportional hazards model with the backward LP method assessed the independent predictors of functionality, and were used to obtain adjusted functional curves. Categorical variables (onset-form and gender) were included as dummy variables. Spinal onset-form and male gender were considered as reference contrast indicators. Functionality was measured either from symptom onset to decay in ALSFRS lower or equal to 0.92/month or censor date (T2), or between the two evaluation periods considering the time interval between them and the same decay in ALSFRS. Continuous variables were not dichotomized. Results were expressed as median [confidence intervals (CI)]. Those meaningful for *p* < 0.05 were evaluated in Cox regression. Analyses were performed in SPSS v20.0 (IBM® SPSS® Statistics).

### Local Ethics' Committee

All procedures were in accordance with the ethical standards of our institutional research committee and with the 1964 Helsinki declaration and its later amendments or comparable ethical standards. This project was approved by the local Ethics' Committee.

## Results

We included 232 ALS patients (134 men; mean onset age was 59.1 ± 11.23 years; body mass index (BMI) at first visit was 25.24 ± 3.6; mean disease duration from symptom onset to first visit was 14.5 ± 12.9 months). Onset form was spinal in 166 patients, bulbar in 66. All patients did SVC, FVC, and ALSFRS at baseline (T1) and a mean of 6.04 ± 1.5 months after (T2). All patients performed FVC and SVC. The functional scores and percentage of predicted values of the respiratory tests are summarized in Table 1.

**Table 1:** Values of the functional scores and percentage of predicted values (%) of the respiratory tests performed at study entry and 6 months after.

	**T1**	**T2**	***p***
ALSFRS	33.76 ± 4.3	28.71 ± 7.2	<0.001^*^
ALSFRSb	10.47 ± 2.2	9.42 ± 3.3	<0.001^*^
ALSFRSul	9.94 ± 2.3	8.2 ± 3.5	<0.001^*^
ALSFRSll	9.63 ± 2.33	7.8 ± 3.2	<0.001^*^
RofALSFRS-R	11.7 ± 0.76	11.0 ± 1.8	<0.001^*^
SVC (%predictive)	94.13 ± 18.7	83.47 ± 19	<0.001^*^
FVC (%predictive)	94.5 ± 19	83.7 ± 24.3	<0.001^*^

* *Meaningful for meaningful for α < 0.05*.

SVC, FVC, ALSFRS, and its subscores decayed significantly (*p* < 0.001) with monthly decays of 0.89 ± 0.91 for ALSFRS, 2.18 ± 2.6% for SVC and 2.23 ± 2.69% for FVC. Monthly decays in the bulbar-onset subgroup were 1.14 ± 0.97 for ALSFRS, 3.05 ± 3.09% for SVC and 3.09 ± 3.3% for FVC while in the spinal-onset subgroup were 0.79 ± 0.87 for ALSFRS, 1.84 ± 2.3% for SVC, and 1.88 ± 2.33% for FVC. Eighty-nine patients had an ALSFRS decay > 0.92/month. Four patients had NIV at T2 but not at T1. These patients presented monthly decays of 0.10 ± 0.19% for SVC, 0.15 ± 0.3% for FVC, and 0.12 ± 0.81 for ALSFRS. No patients had gastrostomy at T1.

SVC and FVC were very strongly correlated with each other at T1 (*r*^2^ = 0.97, *p* < 0.001) and T2 (*r*^2^ = 0.98, *p* < 0.001) for the whole population and similar results were found when considering bulbar and spinal onset patients (all *r*^2^ > 0.93, *p* < 0.001). Correlations with both variables with ALSFRS and RofALSFRS-R were significant (*p* < 0.001) but weak at T1 (ALSFRS: *r*^2^ = 0.24 and *r*^2^ = 0.22; RofALSFRS-R: *r*^2^ = 0.29 and *r*^2^ = 0.22, respectively for SVC and FVC) while moderate at T2 (ALSFRS: *r*^2^ = 0.52 and 0.51; RofALSFRS-R: *r*^2^ = 0.53 and 0.52, respectively for SVC and FVC).

In the Kaplan–Meier analysis, significant predictors of functional decay were onset age (*p* = 0.002), BMI at first visit (*p* < 0.001), disease duration at first visit (*p* < 0.001), ALSFRS, SVC (*p* < 0.001), FVC (*p* < 0.001), SVCdecay (*p* < 0.001), and FVCdecay (*p* < 0.001). Gender, onset form and RofALSFRS-R at study entry were not (*p* > 0.05). Similar results were found when considering spinal and bulbar-onset subgroups.

**Figure 1:**
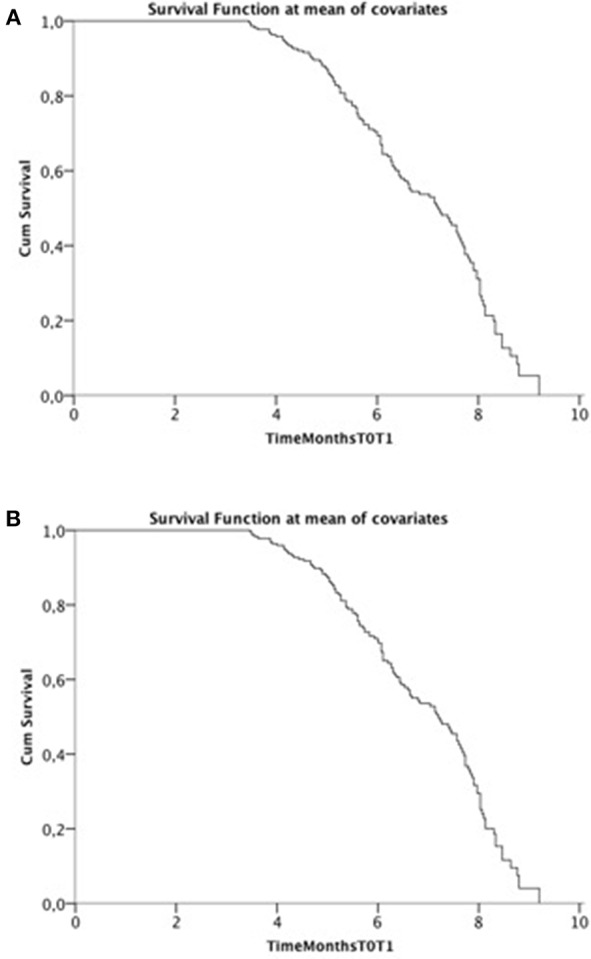
Cox adjusted plot. **(A)** SVC decay; **(B)** FVC decay.

Cox model was run twice including FVC in one and SVC in the other model and their decays (Figure 1). In the final model, including all relevant covariates, only FVCdecay and SVCdecay were independent predictors of functional decay at T2 with similar results (SVC: β = −0.172; exp(β) = 0.842, 95% CI 0.755–0.939, *p* = 0.002; FVCdecay: β = −0.163; exp(β) = 0.85, 95% CI 0.764–0.944, *p* = 0.003). Considering ALSFRS-R and a cut-off of 1.1, similar results were obtained.

## Discussion

With the present study we aimed to investigate how two respiratory function tests commonly used in ALS, SVC, and FVC, could mirror the functional loss in ALS as assessed by the ALSFRS. To minimize the impact of the ventilation support in the global functional assessment of the ALS patients, ALSFRS was selected as the main outcome, not ALSFRS-R. A monthly ALSFRS decay not exceeding 0.92/month was considered as the time event at T2, 6 months after the initial one (T1). This cut-off is equivalent to a 1.1/month decay in ALSFRS-R, accepted to define fast progressors in recent clinical trials (23). By using this cut-off we identified 89 fast-progressing patients. FVCdecay and SVCdecay were significant independent predictors of functional decay, being a lower decay associated with better function at T2, and both showing the same influence. Both FVC and SVC were also highly correlated, this last observation in accordance with two previous studies (19, 20). The 4 patients with NIV placed immediately after T1 had lower functional decays than the mean population (ALSFRS, SVC, and FVC decays). This may be in relation to a floor effect for SVC and FVC, as happens with the ALSFRS (25).

ALSFRS-R has been used worldwide as the main functional outcome scale in ALS. Recently it has been shown that it has its pitfalls, namely regarding the respiratory (26) and the bulbar (27) functional assessments, which can lead to apparent, non-real, reversals in the progression of the disease (28). In fact, a patient with dyspnoea for progressive efforts which culminates in dyspnea during mild efforts or rest, will abstain himself from walking and climbing stairs ending up with cardiovascular, respiratory, and limb deconditioning not depending directly on the dismiss of the motor neurons. On the other hand, a patient with dysphagia and decreased nutritional intake can be markedly debilitated, with dehydration and malnutrition, thereby not having the required energy storages to climb stars, walk, dress, and sometimes, in extreme cases, even to breathe without being dyspnoeic. Nevertheless, it is important to consider that the ALSFRS-R scale is a functional scale and, therefore, improvement in symptomatology by either pharmacological aids (like anti-spastics and antidepressants) and by mechanical aids (like ventilators and PEG) are acceptable and do not represent reversing the neurodegenerative pathophysiological mechanism(s). However, these may impact the outcomes of clinical trials, which use functional scales or tests as outcomes.

This study has some limitations, namely the non-inclusion of other variables that can influence functionality in ALS, apart from NIV and gastrostomy (reasons presented above). Familial/ caregiver support, availability of technical aids (probably nor relevant in our study population), emotional factors and genetic profile were not considered.

To conclude, respiratory functional tests as represented by FVCdecay and SVCdecay are interchangeable as independent factors in predicting functional loss in ALS. Taking into account that SVC and FVC are strongly correlated with each other and with other respiratory measurements, are independent predictors of survival, and their decays are independent predictors of functionality, showing the same magnitude, we suggest that SVC, a more comfortable test to perform, should be preferentially used in clinical practice and in clinical trials for evaluating the respiratory function in ALS. Any pharmacological intervention reducing the decline rates of FVC and SCV should have a positive impact on the global functional impairment, which has relevant implications for clinical trials' design and interpretation of their results.

## Ethics Statement

Project (OnWebDuals) approved by the joint Ethics Committee Faculty of Medicine-Centro Hospitalar Lisboa Norte (Lisbon).

## Author Contributions

All authors listed have made a substantial, direct and intellectual contribution to the work, and approved it for publication.

### Conflict of Interest Statement

The authors declare that the research was conducted in the absence of any commercial or financial relationships that could be construed as a potential conflict of interest.

## Abbreviations

ALS: Amyotrophic Lateral Sclerosis
ALSFRS: ALS functional rating scale
ALSFRS-R: Revised ALSFRS
ALSFRSb: Bulbar subscore of ALSFRSR
FVC: Forced vital capacity
FVCdecay: Decay of FVC
MEP: Maximal expiratory pressure
MIP: Maximal inspiratory pressure
NIV: Non-invasive ventilation
RofALSFRS-R: Respiratory subscore of ALSFRS-R
SVC: Slow vital capacity
SVCdecay: Decay of SVC.

## References

[B1] Federici S Bracalenti M Meloni F Luciano JV. World Health Organization disability assessment schedule 2.0: an international systematic review. Disabil Rehabil. (2017) 39:2347–80. 10.1080/09638288.2016.1223177 27820966

[B2] Cedarbaum JM Stambler N. Performance of the amyotrophic lateral sclerosis functional rating scale (ALSFRS) in multicenter clinical trials. J Neurol Sci. (1997) 152:S1–9. 10.1016/S0022-510X(97)00237-2 9419047

[B3] Cedarbaum JM Stambler N Malta E Fuller C Hilt D Thurmond B The ALSFRSR: a revised ALS functional rating scale that incorporates assessments of respiratory function. BDNF ALS study group (Phase III). J Neurol Sci. (1999) 169:13–21. 10.1016/S0022-510X(99)00210-5 10540002

[B4] Corcia P1 Pradat PF Salachas F Bruneteau G Forestier Nl Seilhean D. Causes of death in a post-mortem series of ALS patients. Amyotroph Lateral Scler. (2008) 9:59–62. 10.1080/17482960701656940 17924236

[B5] EFNS Task Force on Diagnosis and Management of Amyotrophic Lateral Sclerosis Andersen PM Abrahams S Borasio GD de Carvalho M Chio A. EFNS guidelines on the clinical management of amyotrophic lateral sclerosis (MALS)–revised report of an EFNS task force. Eur J Neurol. (2012) 19:360–75. 10.1111/j.1468-1331.2011.03501.x 21914052

[B6] Hutchinson J On the capacity of the lungs, an on the respiratory function, with a view of establishing a precise and easy method of detecting disease by the spirometer. Med Chir Trans. (1846) 29:137–252. 10.1177/095952874602900113 PMC2116876 20895846

[B7] Fitting JW Volitional assessment of respiratory muscle strength. Monaldi Arch Chest Dis. (2012) 77:19–22. 10.4081/monaldi.2012.162 22662641

[B8] Fallat RJ Jewitt B Bass M Kamm B Norris F. Spirometry in amyotrophic lateral sclerosis. Arch Neurol. (1979) 36:74–80. 10.1001/archneur.1979.00500380044004 420626

[B9] Pinto S de Carvalho M. Breathing new life into treatment advances for respiratory failure in amyotrophic lateral sclerosis patients. Neurodegen Dis Manage. (2014) 4:83–102. 10.2217/nmt.13.74 24640982

[B10] Lechtzin N Wiener CM Shade DM Clawson L Diette GB Spirometry in the supine position improves de detection of diaphragmatic weakness in patients with amyotrophic lateral sclerosis. Chest. (2002) 121:436–42. 10.1378/chest.121.2.436 11834654

[B11] Czaplinski A Yen AA Appel SH. Forced vital capacity (FVC) as an indicator of survival and disease progression in an ALS clinic population. J Neurol Neurosurg Psychiatry. (2006) 77:390–2. 10.1136/jnnp.2005.072660 16484652 PMC2077717

[B12] Stambler N Charatan M Cedarbaum JM. Prognostic indicators of survival in ALS. Neurology. (1998) 50:66–72. 10.1212/WNL.50.1.66 9443459

[B13] Armon C Moses D. Linear estimates of rates of disease progression as predictors of survival in patients with ALS entering clinical trials. J Neurol Sci. (1998) 160:S37–41. 10.1016/S0022-510X(98)00196-8 9851647

[B14] Magnus T Beck M Giess R Puls I Naumann M Toyka KV. Disease progression in amyotrophic lateral sclerosis: predictors of survival. Muscle Nerve. (2002) 25:709–14. 10.1002/mus.10090 11994965

[B15] Baumann F Henderson RD Morrison SC Brown M Hutchinson N Douglas JA. Use of respiratory function tests to predict survival in amyotrophic lateral sclerosis. Amyotroph Lateral Scler. (2010) 11:194–202. 10.3109/17482960902991773 19452343

[B16] Pinto S Pinto A de Carvalho M. Phrenic nerve studies predict survival in amyotrophic lateral sclerosis. Clin Neurophysiol. (2012) 123:2454–9. 10.1016/j.clinph.2012.05.012 22762911

[B17] Paillisse C Lacomblez L Dib M Bensimon G Garcia-Acosta S Meininger V. Prognostic factors for survival in amyotrophic lateral sclerosis patients treated with riluzole. Amyotroph Lateral Scler. (2005) 6:37–44. 10.1080/14660820510027035 16036424

[B18] Gil J Preux PM Alioum A Ketzoian C Desport JC Druet-Cabanac M. Disease progression and survival in ALS: First multi-state model approach. Amyotroph Lateral Scler. (2007) 8:224–9. 10.1080/17482960701278562 17653920

[B19] Pinto S de Carvalho M. Correlation between forced vital capacity and slow vital capacity for the assessment of respiratory involvement in Amyotrophic Lateral Sclerosis: a prospective study. Amyotroph Lateral Scler Frontotemporal Degener. (2017) 18:86–91. 10.1080/21678421.2016.1249486 27915482

[B20] Pinto S de Carvalho M. Comparison of slow and forced vital capacities on ability to predict survival in ALS. Amyotroph Lateral Scler Frontotemporal Degener. (2017) 18:528–33. 10.1080/21678421.2017.1354995 28741375

[B21] Roche JC Rojas-Garcia R Scott KM Scotton W Ellis CE Burman R. A proposed staging system for amyotrophic lateral sclerosis. Brain. (2012) 135:847–52. 10.1093/brain/awr351 22271664 PMC3286327

[B22] Miller MR Hankinson J Brusasco V Burgos F Casaburi R Coates A. Standardisation of spirometry. Eur Respir J. (2005) 26:319–38. 10.1183/09031936.05.00034805 16055882

[B23] Mora JS Hermine O AB10015 Study Group. Masitinib as an add-on therapy to riluzole is safe and effective in the treatment of ALS. Amyotroph Lateral Scler Frontotemporal Degener. (2017) 381:183 10.1016/j.jns.2017.08.526

[B24] Kimura F Fujimura C Ishida S Kakajima H Furutama D Uehara H. Progression rate of ALSFRS-R at time of diagnosis predicts survival time in ALS. Neurology. (2006) 66:265–7. 10.1212/01.wnl.0000194316.91908.8a 16434671

[B25] Wicks P Massagli MP Wolf C Heywood J. Measuring function in advanced ALS: validation of ALSFRS-EX extension items. Eur J Neurol. (2009) 16:353–9. 10.1111/j.1468-1331.2008.02434.x 19364363

[B26] Pinto S de Carvalho M. The R of ALSFRS-R: does it really mirror functional respiratory involvement in amyotrophic lateral sclerosis? Amyotroph Lateral Scler Frontotemporal Degener. (2015) 16:120–3. 10.3109/21678421.2014.952641 25205208

[B27] Pinto S Gromicho M de Carvalho M. Sialorrhoea and reversals in ALS functional rating scale. J Neurol Neurosurg Psychiatry. (2017) 88:187–8. 10.1136/jnnp-2016-313614 27634984

[B28] Bedlack RS Vaughan T Wicks P Heywood J Sinani E Selsov R. How common are ALS plateaus and reversals? Neurology. (2016) 86:808–12. 10.1212/WNL.0000000000002251 26658909 PMC4793781

